# Immune System Regulation in the Induction of Broadly Neutralizing HIV-1 Antibodies

**DOI:** 10.3390/vaccines2010001

**Published:** 2013-12-19

**Authors:** Garnett Kelsoe, Laurent Verkoczy, Barton F. Haynes

**Affiliations:** 1 Department of Immunology, Duke University School of Medicine, Durham, NC 27710, USA; 2 The Duke Human Vaccine Institute, Duke University School of Medicine, Durham, NC 27710, USA; 3 Department of Medicine, Duke University School of Medicine, Durham, NC 27710, USA

**Keywords:** HIV-1 vaccines, immune tolerance, broadly neutralizing antibody, B-cell lineage design, HIV-1 vaccine strategy

## Abstract

In this brief review, we discuss immune tolerance as a factor that determines the magnitude and quality of serum antibody responses to HIV-1 infection and vaccination in the context of recent work. We propose that many conserved, neutralizing epitopes of HIV-1 are weakly immunogenic because they mimic host antigens. In consequence, B cells that strongly bind these determinants are removed by the physiological process of immune tolerance. This structural mimicry may represent a significant impediment to designing protective HIV-1 vaccines, but we note that several vaccine strategies may be able to mitigate this evolutionary adaptation of HIV and other microbial pathogens.

## 1. Introduction

While HIV-1 is extraordinarily diverse and mutates rapidly, the HIV-1 envelope has conserved regions to which neutralizing antibodies can be made [1]. Inducing antibodies by HIV-1 vaccine candidates is a major goal of HIV-1 vaccine development. However, repeated attempts at HIV-1 envelope immunization of animals and man have induced primarily non-neutralizing or HIV-1 strain-specific antibodies. In this review, we were charged to discuss our recent work in the area of host control of humoral responses to HIV-1; consequently, we have not included much outstanding work by the many other investigators who have contributed to understanding humoral immunity to HIV-1. We shall, nonetheless, point out some of the roadblocks that are currently hindering the successful induction of broadly reactive neutralizing antibodies (bnAbs) to the HIV-1 envelope.

## 2. HIV-1 Envelope Antigenicity and Immunogenicity

Not all HIV-1 envelope epitopes are created equal: some are highly immunogenic and dominate antibody (Ab) responses, whereas others are weakly immunogenic and are sub-dominant [2]. Significantly, the neutralizing epitopes that are shared by multiple HIV-1 clades and elicit bnAb responses are weakly immunogenic and elicit little or no protective immunity in most infected patients [3]. A key question that has occupied the HIV-1 field for the last 5 years is “why are BnAbs so difficult to induce?”

Most theories to explain the variability of HIV-1 Env immunogenicity focus on limitations of the antigen-receptor repertoire [4,5,6], epitope shielding by interfering structures [7,8,9], and inflammatory capacity [10,11,12]. Evidence to support each of these hypotheses exist but until recently there has been no concerted effort to focus immune responses on subdominant epitopes to exploit their potential utility as vaccine antigens [2,13,14,15,16,17,18,19]. 

The properties of immunogenicity and antigenicity are related but distinct: immunogens are capable of inducing humoral or cell-mediated, adaptive immune responses while antigens are structures that specifically bind to the antigen receptors of B- or T cells (BCR or TCR, respectively). Consequently, immunogens are always antigens but antigens are not necessarily immunogenic. The classical example of this dichotomy is the hapten; haptens such as nitrophenyl(acetyl) are bound by BCR and antibodies with great specificity but alone cannot elicit humoral responses.

The dissociation of antigenicity and immunogenicity of HIV-1 envelope is the essence of the problem in eliciting bnAb responses. That is, while the HIV-1 envelope gp120 and gp41 components have conserved, neutralizing epitopes that are *antigenic*, *i.e*., rare antibodies can indeed bind to Env proteins, they are not *immunogenic* and do not induce neutralizing antibodies targeted at these sites. Largely, epitopes are identified by elicited antibodies, *i.e*., epitopes that are both antigenic and immunogenic. For most antigens, this dual definition is not problematic. However, for the conserved HIV-1 antigens that do not elicit protective immune responses, understanding immunogenicity is crucial. For example, the bnAb epitopes in the membrane-proximal external region (MPER) of envelope gp41 can be mimicked by scaffolds and peptide-liposome immunogens that induce antibodies to bind precisely at the bnAb polypeptide epitope [13,20]. However, scaffold-induced Ab does not neutralize HIV-1 and the epitope-specific component of the serum response is only a small minority of the induced Ab. We conclude that at least some HIV-1 epitopes are intrinsically weak immunogens.

The role of immune tolerance in the weak immunogenicity of neutralizing MPER epitopes is challenged by the discovery of the 10E8 bnAb [21]. This bnAb recognizes an epitope that overlaps the conserved 4E10 MPER determinant and exhibits great neutralizing breadth [98% (178/181) of HIV-1 isolates tested] *in vitro*. The 10E8 bnAb was reported to have no affinity for phospholipids, including phosphatidyl choline-cardiolipin, did not label fixed HEp-2 human epithelia cells, nor did it bind several human autoantigens diagnostic for various autoimmune diseases [21]. Nonetheless, in a protein microarray [3], whereas 10E8 was observed not to be polyreactive, it did exhibit strong affinity for a human protein expressed in variety of mature organs as well as in fetal tissues [22]. Whether this autoreactivity is physiologically significant remains to be determined by the generation of 10E8 knockin mice [23,24,25].

HIV-1 bnAb also recognize complex epitopes. For example, the 2F5 and 4E10 bnAb bind MPER determinants composed both of membrane lipid and the gp41 polypeptide [26]. In addition, these MPER bnAb epitopes mimic host antigens [3,27] such that immune tolerance mechanisms deplete or anergize reactive B cells [23,24,25,28,29]. Thus, vaccine immunogens that induce HIV-1 bnAbs may necessarily have complex structure (e.g., lipid and polypeptide) and/or be formulated to overcome host immune control mechanisms.

## 3. Designing New Vaccine Strategies

### 3.1. HIV-1 Broadly Neutralizing Antibodies

HIV-1 bnAbs have unusual but characteristic features, most notably, extraordinary frequencies of V(D)J mutations, that imply unusual developmental histories [2,30]. The frequencies of mutations characteristic of bnAbs include point mutations and insertions and deletions (indels). Point mutations from high levels of somatic hypermutation (SHM) range from 15% to >30%, and demand that we consider why high levels of SHM appear necessary for bnAb production [31]. In addition, a substantial number of bnAbs appear to be poly- and/or autoreactive [2]. For a thorough guide to the origins and characteristics of bnAbs, we refer our readers to a recent review [1] that includes a summary table of 22 bnAbs identified between 1993–2013. 

In germinal centers (GC), antigen-reactive mature B cells proliferate and express high amounts of activation-induced cytosine deaminase (AID), an enzyme required for immunoglobulin class-switch recombination and *V(D)J* hypermutation [32]. The clonal evolution of GC B cells is a Darwinian process comprising two mechanisms: SHM and affinity-dependent selection. Selection vets GC B cell populations for increased affinity for the immunogen [33,34]. Indeed, GC B cell survival and proliferation is determined by BCR affinity and the capacity of each B cell to collect and present antigen to local GC T-helper (T_FH_) cells [35].

Clonal selection in GCs depends on relative BCR fitness (affinity and specificity) and changes over the course of the immune response as novel V(D)J mutations exert their effects. A GC represents an “experiment” in clonal evolution with regard to the founding B- and T-cell populations and the order and distribution of the introduced V(D)J mutations. A conundrum of bnAb development is how GC B cells could acquire mutation frequencies of 15%–30% while maintaining their ability to bind antigen and effectively compete for T_FH_ help. In general, as mutant BCR fitness (affinity) increases, it becomes increasingly likely that additional mutations are maladaptive. Reduced BCR fitness in GC leads to rapid clonal elimination [36,37] and there is no reason to believe that the capacity to neutralize multiple HIV-1 clades—or to neutralize at all—provides any selective advantage to GC B cells.

At least three hypotheses are currently proposed to explain the high mutation frequencies of HIV-1 bnAb. First, that these mutations are necessary to modify germline Abs so as to meet unusually stringent structural requirements. These structural requirements might include not only high affinity, but also restriction to a core epitope that is poorly recognized by the primary, germline, Ab repertoire [31]. Two alternative hypothesis are influenced by the observations of frequent poly- and/or autoreactivity among bnAbs [2,30]. One alternative hypothesis is that many (most?) conserved HIV-1 neutralizing epitopes have been selected to mimic host antigens; consequently, bnAbs are heavily mutated because the germline Ab/BCR that best recognize these epitopes are lost to immunological tolerance. In this model, affinity maturation for a neutralizing epitope represents *de novo* mutation and selection acting on weakly cross-reactive, previously mutated B cells [2,3,23,30]. Another, related and non-exclusive possibility is that the structural overlap between HIV-1 neutralization- and host epitopes is close but not complete. In this case, mutated, anergic B-cells with neutralization activity undergo virus-driven, “conflicted” purifying selection that acts on V(D)J residues that remove self-reactivity while maintaining affinity for the neutralization epitope [28]. Autoreactivity has been shown to increase in the human GC B cell compartment as a result of V(D)J mutations that alter antibody specificity [38].

### 3.2. Wolves in Sheep’s Clothing

Infection by HIV-1 poses a remarkable immunological conundrum: conserved neutralizing epitopes are present on HIV-1 envelope (Env) but rarely elicit protective Ab. The unusual, shared traits of bnAbs suggest an atypical clonal evolution that would normally decrease, not enhance, B-cell fitness [2]; they almost certainly represent the efforts of the immune system to both respond to weakly immunogenic neutralizing epitopes, while avoiding producing antibodies with the polyreactivity, long heavy chain complementarity determining (HCDR3) regions and high levels of SHMs.

At least one evolutionary strategy used by pathogens to moderate immunogenicity is host mimickry; immunological tolerance can limit or prevent the production of Ab against microbial epitopes that mimic host structures [30]. For example, Ab elicited by bacterial adhesin FimH of fimbriated pathogens cross-reacts with lysosomal membrane protein-2 (LAMP-2) and causes pauci-immune focal necrotizing glomerulonephritis (FNGN) [39]. Similarly, *Campylobacter jejuni* lipooligosaccharide (LOS) shares epitopes with mammalian, neuronal gangliosides [40], a mimickry associated with modest Ab responses in a minority of infected patients [41]. Immunization of normal mice with *C. jejuni* LOS elicits weak, T-dependent Ab responses but these are greatly enhanced in mice unable to generate complex gangliosides [41].

More recently, we and our colleagues have demonstrated that HIV-1 Ab responses to two highly conserved, neutralizing epitopes of the gp41 MPER of HIV-1 are suppressed as a consequence of immunological tolerance. The 2F5 and 4E10 epitopes of HIV-1 exhibit significant structural similarity to proteins present in most mammals and the B cells that recognize these shared determinants are lost during their development [3,23,24]. Briefly, Verkoczy *et al*. has generated knock-in mice that carry the V(D)J rearrangements of the 2F5 or 4E10 bnAbs; these mice support robust early B-cell development, but exhibit a characteristic block at the small pre-B to immature B cell transition that defines the first tolerance check-point [23,24,38]. This developmental blockade is also present in mouse lines carrying the unmutated, *i.e*., germline 2F5 V(D)J rearrangements, that were expressed by the naïve B cells that gave rise to the high-affinity, mutated 2F5 MPER bnAbs [42].

Our studies in knock-in animals defined the mutated and germline 2F5 V(D)J rearrangements as sufficiently autoreactive to trigger physiologic tolerance, but they did not define the self-antigens mimicked by these MPER epitopes [23,24,28]. Using traditional immunoprecipitation and protein microarrays we identified two highly conserved host self-antigens that were avidly recognized by the 2F5 and 4E10 bnAbs: kynureninase (Kynu) and splice factor 3B3 (SF3B3), respectively [3]. Kynu and SF3B3 effectively inhibit the binding of the 2F5 and 4E10 bnAbs to their HIV-1 MPER epitopes; in the case of Kynu, this inhibition is mediated by amino acid identity between the HIV and host protein [3]. A larger survey of HIV-1 bnAbs by these same methods [22] indicates that mimicry of host antigens is common, supporting the hypothesis that viral evolution favors structural similarity with host proteins as a way to mitigate immune responses that diminish transmission [30].

Microbial mimicry of host antigens is an effective strategy to mitigate humoral immunity in the infected host. We propose that this evolutionary strategy is more widespread than currently recognized and a principal component of weak neutralizing/protective Ab responses to key microbial epitopes. Indeed, one of the central issues of host-pathogen biology has been whether, or to what extent, self-tolerance limits the B- and T-cell repertoires available for responses to pathogens. Work from the Nussenzweig laboratory has shown that in humans, approximately half of the primary naïve B cell repertoire is lost to the first and second tolerance checkpoints [38,43]. It would be surprising if such substantial losses did not reduce the capacity to react with viruses and other microbes, but the degree to which this happens is not known. If tolerance substantially reduces the antibody repertoire available for protective immunity to pathogens, a new world of (potentially useful) epitopes is hidden by immune tolerance.

### 3.3. Physiological B-Cell Tolerance

B cells develop from lineage-specific progenitors that express the V(D)J recombinase [44] and first rearrange the immunoglobulin heavy locus (IGH) gene loci to generate a pre-B cell receptor (pre-BCR). The pre-BCR do not bind antigens but their assembly is necessary for continued cell survival and proliferation. Pre-B cells exit the cell cycle as pre-B II cells, initiate rearrangements in the κ or λ light-chain loci and assemble a mature BCR that binds antigen. The generation of BCRs by combinatiorial association of V (variable), D (diversity) and J (joining) gene segments generates a diverse primary repertoire of BCRs but frequently produces self-reactive B cells [38,43].

Indeed, most newly generated, or immature B cells in the bone marrow are autoreactive and must be eliminated or inactivated by immunological tolerance. The remaining B cells mature through the transitional 1 (T1) and T2 stages, which are characterized by changes in membrane IgM and IgD expression and the loss or diminution of markers associated with developmental immaturity. In the periphery, newly formed (T2) B cells are subject to a second round of immune tolerization before entering the mature B-cell pools.

Three mechanisms of immunological tolerance are known to deplete B-cell pools of self-reactivity: apoptosis, cellular inactivation by anergy and replacement of autoreactive BCRs by secondary *V(D)J* rearrangement [45,46]. The majority of lymphocytes committed to the B-cell lineage do not reach maturity as they do not express functional µH polypeptides or because they carry self-reactive BCRs [47,48].

Autoreactive B cell numbers decline with increasing B-cell maturity. Tolerance mechanisms, especially apoptotic deletion, operate during the transitional stages of B-cell development, and the number of self-reactive cells decreases substantially after entry into the mature pools. Nonetheless, not all autoreactive B cells are lost; some 20%–25% of mature, naive B cells circulating in human blood express autoreactive BCRs [38,43].

The corollary to our proposal that the infrequency of bnAbs production is due—at least in part—to the deletion/inactivation of HIV-1 specific B cells that acquire autoreactivity [3,23,24,25,27] is that HIV-1 infected subjects with autoimmune diseases might be more capable of developing bnAbs [30]. Observations that subjects with systemic lupus erythematosus (SLE) and HIV-1 infection are reported at disproportionately low frequencies support this hypothesis [49,50,51,52] but to date, no direct evidence on this point has been published.

### 3.4. Looking Backward

Pathways leading to bnAb generation have been traced by following the evolution of B-cell lineages back to their origins, the unmutated, mature IgM^+^IgD^+^ B cells that first responded to virus infection [30,53,54]. Can these recently discovered pathways define a method for the generation of protective Ab responses by vaccines?

Current HIV-1 vaccines can elicit strain-specific neutralizing Ab but not bnAb; bnAb do arise, however, in approximately 20% of HIV-1-infected individuals, albeit after years of infection [55,56,57,58]. These uncommon bnAb responders provide an opportunity to follow the bnAb response backwards, in effect, reversing B-cell evolution to identify antigen-ligands that might re-create this (or comparable) bnAb pathways in many individuals [2,59,60].

Harnessing recent advances in flow cytometry, viral genomics, human B-cell culture, recombinant antibody technology, 454 deep sequencing, and bioinformatics, Liao *et al*. [54] recently demonstrated the co-evolution of HIV-1 and a bnAb B-cell lineage that produced Ab directed to the virus CD4 binding-site. Sequence analysis of virus and antibody V(D)J genes successively recovered from an infected patient revealed that the unmutated B-cell ancestor of this bnAb lineage strongly reacted with Env of the transmitted/founder virus. This ancestral B cell did not exhibit bnAb activity but the evolution of neutralization breadth increased over time and was associated with viral diversification suggesting immune-mediated selection at the neutralizing epitope [54]. 

**Figure 1:**
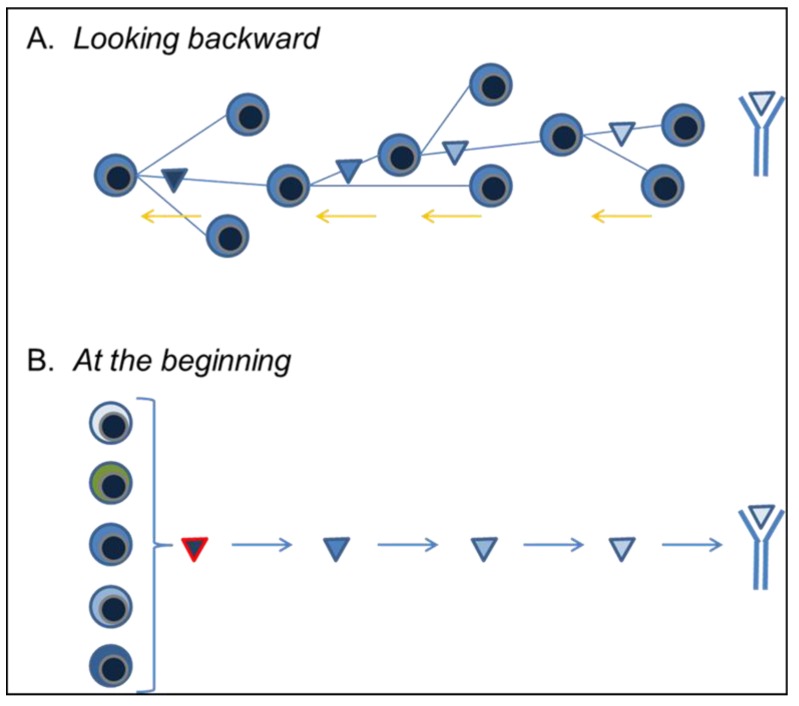
Synopsis of proposed vaccine strategies. (**A**) The B-lineage vaccine approach to eliciting HIV-1 bnAb responses in vaccinees is based on the recapitulation of clonal evolutionary pathways that lead to bnAb in a single HIV infected patient [2,54]. This approach relies on the technical capacity to identify, recover, and characterize bnAb B cells and/or plasmacytes from HIV-1 infected patients. BCR encoding gene rearrangements isolated from single bnAb cells are then used to to follow the bnAb response backwards, in effect, reversing B-cell evolution to identify antigen-ligands that might re-create this (or comparable) bnAb pathways in many individuals [2]. In this way, vaccine ligands can be generated and optimized to select for the desired—bnAb—evolutionary pathway; (**B**) The ancillary approach is us to identify those cells in the naïve B-cell pools capable of recognizing HIV-1 neutralizing epitopes and/or optimized, lineage design, vaccine immunogens. In this approach, cultured B cells are driven to proliferate by exposure to CD154 and immunogens capable of driving bnAb production can be modified to activate the largest possible pool of specific B cells. In consequence, we can study HIV-1 antigen epitopes without regard to their ability to elicit significant humoral responses: antigenicity minus immunogenicity.

Together, the bnAb and virus sequence data assembled by Liao and his colleagues [54] describe the viral and Ab/B-cell evolution culminating in bnAb production. Importantly, the evolving, mutant BCR of the bnAb lineage could be shown to react strongly with successive HIV-1 Env mutants. This shared pattern of reactivity, immediately provided a starting point for Env vaccine constructs to induce reproducibly bnAb generation [2,53] (Figure 1A).

Interestingly, Liao *et al*. [54] observed that intense virus selection and diversification preceded the development of bnAb activity and that neutralization breadth was associated with the acquisition of poly- and autoreactivity. These findings imply that serial vaccine immunogens optimized for binding to the founders and intermediates of bnAb lineages [2] would likely mimic the natural pathways for bnAb development and be capable of overcoming the limiting effects of immune tolerance on bnAb generation. If so, this novel immunization approach offers a logical strategy for inducing B-cell evolution along rare bnAb pathways that cannot be elicited by conventional, single-immunogen vaccines.

### 3.5. At the Beginning

As an adjunct to the use of serial, optimized immunogens to elicit known bnAb pathways, we have developed culture systems [60,61] for naïve B cells that permit the dissociation of antigenicity and immunogenicity. In brief, we culture single B cells under conditions that support extensive cell proliferation and efficient plasmacytic differentiation [62]. The specificity of Ab secreted into each clonal culture can be screened rapidly in multiplex assays to determine specificity and avidity; the new recombinant Ab technology makes it a simple matter to generate larger quantities of Ab for neutralization studies. Perhaps most significantly, in addition to mature IgM^+^IgD^+^ B cells, we can culture late pre-B cells, immature/transitional (imm/T) B cells, GC and memory B cells, and “anergic” B cells [24,28,61]. We can, therefore, identify and characterize the primary B-cell repertoire before and after the first- and second tolerance checkpoints to determine their role in regulating HIV-1 bnAb production.

These cultures allow us to identify and study (in mice and humans) the potential B-cell repertoire—*i.e*., before the tolerance checkpoints—capable of recognizing HIV-1 neutralizing epitopes and to contrast that with the post-tolerance, expressed repertoire that initiates humoral responses. Cultured B cells are driven to proliferate by exposure to CD154 (CD40L), and although BCR expression is necessary for cell survival, activation by epitope ligands is unnecessary. In consequence, we can study HIV-1 antigen epitopes without regard to their ability to elicit significant humoral responses: antigenicity minus immunogenicity.

We are using this novel technology to identify and characterize unmutated BCR/Ab that recognize neutralizing HIV-1 epitopes in the gp41 MPER and gp120 CD4 binding site. The biology and structures of these Env epitopes are exceptionally well characterized and available as highly purified recombinant proteins. We plan on screening vaccine immunogens that initiate BnAb lineages (Figure 1A) for their capacity to react with the broadest possible subset of naïve B cells (Figure 1B). In this way, engineered B-cell lineage immunogens [2] can have the widest possible impact in genetically diverse vaccine populations.

## 4. Moving Forward

The long delayed appearance and infrequency of HIV-1 bnAb production has re-opened one of immunology’s central questions: to what extent does self-tolerance impact immunity to pathogens? It has been widely assumed that self and foreign epitopes are virtually non-overlapping and that whereas tolerance may remove some pathogen-specific B cells, epitope coverage is not significantly affected. We and others have now shown that, at least for some HIV-1 neutralizing epitopes, this is not the case: the overlap between foreign and self-antigens can be significant and has substantial impact on protective immunity. This demonstration opens the way to new vaccine strategies. We note that the host-mimicry by microbial epitopes is not necessarily a barrier to using them in vaccines [2,54,63].

The simplest form of a lineage-based vaccine design [2], is offered by the recent work of Liao and colleagues [54]. In this example, a candidate vaccine could be constructed by using serial isolates of mutant HIV-1 Env to drive the evolutionary intermediates of a known bnAb lineage. Whereas this vaccine strategy would be very likely to work in the same infected individual, whether it would be equally effective in genetically dissimilar vaccines remains a crucial question. Nonetheless, the use of a known T/F Env to activate naïve bnAb B-cell ancestors followed by booster immunizations specific, mutated Env variants is an attractive approach to direct the BCR evolution along a pathway that leads to bnAb dominance [2]. Characterization of the primary B-cell repertoire will allow these selected immunogens to be engineered for the broadest possible reactivity that remains consistent with bnAb evolution. This approach will maximize vaccine effectiveness in diverse populations and may even identify naïve B cells that can produce bnAb sooner or with fewer V(D)J mutations.

A variation on the theme to the idea of lineage-based vaccines based on established natural histories of bnAb responses [2,54], is the design of HIV-1 immunogens designed to interact with specific antigen-receptors on naïve B cells [17,64]. This rational approach to immunogen design is analogous to that used to develop or modify drugs and depends on the remarkable wealth of structural data available for bnAb and their neutralizing epitopes [17]. It has in common with B cell lineage design the targeting of unmutated ancestor antibodies of bnAb lineages [2]. At present, the high specificity of this approach is both a strength and a weakness: nanoparticles bearing the CD4 binding site epitope recognized by the VRC01 bnAb [64] activate B cell lines expressing the VRC01 Ab and its inferred germline counterpart, but as mice, rabbits, and rhesus macaques lack V_H_ gene segments capable of interacting with the immunogen, the utility of these vaccines has not yet been determined in immunization studies [17,64]. 

## 5. Conclusions

It is now clear that weakly immunogenic, but powerfully neutralizing epitopes exist not just on HIV-1 [65] but also on pathogens as familiar as influenza [66,67] and *C. jejuni* [41]. We propose that these potentially useful, cryptic epitopes are common on microbial antigens and can offer new and useful targets for vaccine development. The nature of these weak immunogens demand, however, novel vaccine strategies and new ways of thinking about immune activation and high-affinity antibody selection.

## References

[1] Mascola J.R., Haynes B.F. (2013). HIV-1 neutralizing antibodies: Understanding nature’s pathways. Immunol. Rev..

[2] Haynes B.F., Kelsoe G., Harrison S.C., Kepler T.B. (2012). B-cell-lineage immunogen design in vaccine development with HIV-1 as a case study. Nat. Biotechnol..

[3] Yang G., Holl T.M., Liu Y., Li Y., Lu X., Nicely N.I., Kepler T.B., Alam S.M., Liao H.X., Cain D.W. (2013). Identification of autoantigens recognized by the 2F5 and 4E10 broadly neutralizing HIV-1 antibodies. J. Exp. Med..

[4] Gorny M.K., Stamatatos L., Volsky B., Revesz K., Williams C., Wang X.H., Cohen S., Staudinger R., Zolla-Pazner S. (2005). Identification of a new quaternary neutralizing epitope on human immunodeficiency virus type 1 virus particles. J. Virol..

[5] Hicar M.D., Kalams S.A., Spearman P.W., Crowe J.E. (2010). Emerging studies of human HIV-specific antibody repertoires. Vaccine.

[6] Briney B.S., Willis J.R., Crowe J.E. (2012). Human peripheral blood antibodies with long HCDR3s are established primarily at original recombination using a limited subset of germline genes. PLoS One.

[7] Wyatt R., Kwong P.D., Desjardins E., Sweet R.W., Robinson J., Hendrickson W.A., Sodroski J.G. (1998). The antigenic structure of the HIV gp120 envelope glycoprotein. Nature.

[8] Kwong P.D., Doyle M.L., Casper D.J., Cicala C., Leavitt S.A., Majeed S., Steenbeke T.D., Venturi M., Chaiken I., Fung M. (2002). HIV-1 evades antibody-mediated neutralization through conformational masking of receptor-binding sites. Nature.

[9] Wei X., Decker J.M., Wang S., Hui H., Kappes J.C., Wu X., Salazar-Gonzalez J.F., Salazar M.G., Kilby J.M., Saag M.S. (2003). Antibody neutralization and escape by HIV-1. Nature.

[10] Van Duin D., Medzhitov R., Shaw A.C. (2006). Triggering TLR signaling in vaccination. Trends Immunol..

[11] Palm N.W., Medzhitov R. (2009). Immunostimulatory activity of haptenated proteins. Proc. Natl. Acad. Sci. USA.

[12] Palm N.W., Medzhitov R. (2009). Pattern recognition receptors and control of adaptive immunity. Immunol. Rev..

[13] Ofek G., Guenaga F.J., Schief W.R., Skinner J., Baker D., Wyatt R., Kwong P.D. (2010). Elicitation of structure-specific antibodies by epitope scaffolds. Proc. Natl. Acad. Sci. USA.

[14] Azoitei M.L., Correia B.E., Ban Y.E., Carrico C., Kalyuzhniy O., Chen L., Schroeter A., Huang P.S., McLellan J.S., Kwong P.D. (2011). Computation-guided backbone grafting of a discontinuous motif onto a protein scaffold. Science.

[15] Guenaga J., Dosenovic P., Ofek G., Baker D., Schief W.R., Kwong P.D., Karlsson Hedestam G.B., Wyatt R.T. (2011). Heterologous epitope-scaffold prime:boosting immuno-focuses B cell responses to the HIV-1 gp41 2F5 neutralization determinant. PLoS One.

[16] Zolla-Pazner S., Kong X.P., Jiang X., Cardozo T., Nadas A., Cohen S., Totrov M., Seaman M.S., Wang S., Lu S. (2011). Cross-clade HIV-1 neutralizing antibodies induced with V3-scaffold protein immunogens following priming with gp120 DNA. J. Virol..

[17] Jardine J., Julien J.P., Menis S., Ota T., Kalyuzhniy O., McGuire A., Sok D., Huang P.S., MacPherson S., Jones M. (2013). Rational HIV immunogen design to target specific germline B cell receptors. Science.

[18] Schiffner T., Kong L., Duncan C.J., Back J.W., Benschop J.J., Shen X., Huang P.S., Stewart-Jones G.B., Destefano J., Seaman M.S. (2013). Immune focusing and enhanced neutralization induced by HIV-1 gp140 chemical cross-linking. J. Virol..

[19] McGuire A.T., Hoot S., Dreyer A.M., Lippy A., Stuart A., Cohen K.W., Jardine J., Menis S., Scheid J.F., West A.P. (2013). Engineering HIV envelope protein to activate germline B cell receptors of broadly neutralizing anti-CD4 binding site antibodies. J. Exp. Med..

[20] Dennison S.M., Sutherland L.L., Jaeger F., Anasti K., Parks R., Stewart S.M., Bowman C., Xia S., Zhang R., Shen X. (2011). Induction of antibodies in rhesus macaques that recognize a fusion-intermediate conformation of HIV-1 gp41. PLoS One.

[21] Huang J., Ofek G., Laub L., Louder M.K., Doria-Rose N.A., Longo N.S., Imamichi H., Bailer R.T., Chakrabarti B., Sharma S.K. (2012). Broad and potent neutralization of HIV-1 by a gp41-specific human antibody. Nature.

[22] Liu M., Yang G., Cain D., Wiehe K., Nicely N., Gao J., Haynes B.F., Conners M.J., Mascola J.R., Bjorkman P. (2013).

[23] Verkoczy L., Diaz M., Holl T.M., Ouyang Y.B., Bouton-Verville H., Alam S.M., Liao H.X., Kelsoe G., Haynes B.F. (2010). Autoreactivity in an HIV-1 broadly reactive neutralizing antibody variable region heavy chain induces immunologic tolerance. Proc. Natl. Acad. Sci. USA.

[24] Verkoczy L., Chen Y., Bouton-Verville H., Zhang J., Diaz M., Hutchinson J., Ouyang Y.B., Alam S.M., Holl T.M., Hwang K.K. (2011). Rescue of HIV-1 broad neutralizing antibody-expressing B cells in 2F5 VH × VL knockin mice reveals multiple tolerance controls. J. Immunol..

[25] Chen Y., Zhang J., Hwang K.K., Bouton-Verville H., Xia S.M., Newman A., Ouyang Y.B., Haynes B.F., Verkoczy L. (2013). Common tolerance mechanisms, but distinct cross-reactivities associated with gp41 and lipids, limit production of HIV-1 broad neutralizing antibodies 2F5 and 4E10. J. Immunol..

[26] Alam S.M., Morelli M., Dennison S.M., Liao H.X., Zhang R., Xia S.M., Rits-Volloch S., Sun L., Harrison S.C., Haynes B.F. (2009). Role of HIV membrane in neutralization by two broadly neutralizing antibodies. Proc. Natl. Acad. Sci. USA.

[27] Haynes B.F., Fleming J., St Clair E.W., Katinger H., Stiegler G., Kunert R., Robinson J., Scearce R.M., Plonk K., Staats H.F. (2005). Cardiolipin polyspecific autoreactivity in two broadly neutralizing HIV-1 antibodies. Science.

[28] Verkoczy L., Chen Y., Zhang J., Bouton-Verville H., Newman A., Lockwood B., Scearce R.M., Montefiori D.C., Dennison S.M., Xia S.M. (2013). Induction of HIV-1 broad neutralizing antibodies in 2F5 knock-in mice: Selection against membrane proximal external region-associated autoreactivity limits T-dependent responses. J. Immunol..

[29] Doyle-Cooper C., Hudson K.E., Cooper A.B., Ota T., Skog P., Dawson P.E., Zwick M.B., Schief W.R., Burton D.R., Nemazee D. (2013). Immune tolerance negatively regulates B cells in knock-in mice expressing broadly neutralizing HIV antibody 4E10. J. Immunol..

[30] Haynes B.F., Moody M.A., Verkoczy L., Kelsoe G., Alam S.M. (2005). Antibody polyspecificity and neutralization of HIV-1: A hypothesis. Hum. Antib..

[31] Klein F., Diskin R., Scheid J.F., Gaebler C., Mouquet H., Georgiev I.S., Pancera M., Zhou T., Incesu R.B., Fu B.Z. (2013). Somatic mutations of the immunoglobulin framework are generally required for broad and potent HIV-1 neutralization. Cell.

[32] Di Noia J.M., Neuberger M.S. (2007). Molecular mechanisms of antibody somatic hypermutation. Annu. Rev. Biochem..

[33] Dal Porto J.M., Haberman A.M., Kelsoe G., Shlomchik M.J. (2002). Very low affinity B cells form germinal centers, become memory B cells, and participate in secondary immune responses when higher affinity competition is reduced. J. Exp. Med..

[34] Shih T.A., Meffre E., Roederer M., Nussenzweig M.C. (2002). Role of BCR affinity in T cell dependent antibody responses *in vivo*. Nat. Immunol..

[35] Victora G.D., Schwickert T.A., Fooksman D.R., Kamphorst A.O., Meyer-Hermann M., Dustin M.L., Nussenzweig M.C. (2010). Germinal center dynamics revealed by multiphoton microscopy with a photoactivatable fluorescent reporter. Cell.

[36] Jacob J., Kelsoe G. (1992). *In situ* studies of the primary immune response to (4-hydroxy-3-nitrophenyl)acetyl. II. A common clonal origin for periarteriolar lymphoid sheath-associated foci and germinal centers. J. Exp. Med..

[37] Jacob J., Przylepa J., Miller C., Kelsoe G. (1993). *In situ* studies of the primary immune response to (4-hydroxy-3-nitrophenyl)acetyl. III. The kinetics of V region mutation and selection in germinal center B cells. J. Exp. Med..

[38] Wardemann H., Nussenzweig M.C. (2007). B-cell self-tolerance in humans. Adv. Immunol..

[39] Kain R., Exner M., Brandes R., Ziebermayr R., Cunningham D., Alderson C.A., Davidovits A., Raab I., Jahn R., Ashour O. (2008). Molecular mimicry in pauci-immune focal necrotizing glomerulonephritis. Nat. Med..

[40] Yu R.K., Ariga T., Usuki S., Kaida K. (2011). Pathological roles of ganglioside mimicry in Guillain-Barre syndrome and related neuropathies. Adv. Exp. Med. Biol..

[41] Bowes T., Wagner E.R., Boffey J., Nicholl D., Cochrane L., Benboubetra M., Conner J., Furukawa K., Furukawa K., Willison H.J. (2002). Tolerance to self gangliosides is the major factor restricting the antibody response to lipopolysaccharide core oligosaccharides in *Campylobacter jejuni* strains associated with Guillain-Barre syndrome. Infect. Immun..

[42] Verkoczy L., Bouton-Verville H., Diaz M., Haynes B.F. (2013).

[43] Wardemann H., Yurasov S., Schaefer A., Young J.W., Meffre E., Nussenzweig M.C. (2003). Predominant autoantibody production by early human B cell precursors. Science.

[44] Lai A.Y., Kondo M. (2006). Asymmetrical lymphoid and myeloid lineage commitment in multipotent hematopoietic progenitors. J. Exp. Med..

[45] Nemazee D.A., Burki K. (1989). Clonal deletion of B lymphocytes in a transgenic mouse bearing anti-MHC class I antibody genes. Nature.

[46] Erikson J., Radic M.Z., Camper S.A., Hardy R.R., Carmack C., Weigert M. (1991). Expression of anti-DNA immunoglobulin transgenes in non-autoimmune mice. Nature.

[47] Melchers F. (2005). The pre-B-cell receptor: Selector of fitting immunoglobulin heavy chains for the B-cell repertoire. Nat. Rev. Immunol..

[48] Melchers F., Rolink A.R. (2006). B cell tolerance—How to make it and how to break it. Curr. Top. Microbiol. Immunol..

[49] Barthel H.R., Wallace D.J. (1993). False-positive human immunodeficiency virus testing in patients with lupus erythematosus. Semin. Arthritis Rheum..

[50] Mylonakis E., Paliou M., Greenbough T.C., Flaningan T.P., Letvin N.L., Rich J.D. (2000). Report of a false-positive HIV test result and the potential use of additional tests in establishing HIV serostatus. Arch. Intern. Med..

[51] Palacios R., Santos J., Valdivielso P., Marquez M. (2002). Human immunodeficiency virus infection and systemic lupus erythematosus. An unusual case and a review of the literature. Lupus.

[52] Calza L., Manfredi R., Colangeli V., D’Antuono A., Passarini B., Chiodo F. (2003). Systemic and discoid lupus erythematosus in HIV-infected patients treated with highly active antiretroviral therapy. Int. J. STD AIDS.

[53] Bonsignori M., Hwang K.K., Chen X., Tsao C.Y., Morris L., Gray E., Marshall D.J., Crump J.A., Kapiga S.H., Sam N.E. (2011). Analysis of a clonal lineage of HIV-1 envelope V2/V3 conformational epitope-specific broadly neutralizing antibodies and their inferred unmutated common ancestors. J. Virol..

[54] Liao H.X., Lynch R., Zhou T., Gao F., Alam S.M., Boyd S.D., Fire A.Z., Roskin K.M., Schramm C.A., Zhang Z. (2013). Co-evolution of a broadly neutralizing HIV-1 antibody and founder virus. Nature.

[55] Gray E.S., Madiga M.C., Moore P.L., Mlisana K., Abdool Karim S.S., Binley J.M., Shaw G.M., Mascola J.R., Morris L. (2009). Broad neutralization of human immunodeficiency virus type 1 mediated by plasma antibodies against the gp41 membrane proximal external region. J. Virol..

[56] Gray E.S., Taylor N., Wycuff D., Moore P.L., Tomaras G.D., Wibmer C.K., Puren A., DeCamp A., Gilbert P.B., Wood B. (2009). Antibody specificities associated with neutralization breadth in plasma from human immunodeficiency virus type 1 subtype C-infected blood donors. J. Virol..

[57] Gray E.S., Madiga M.C., Hermanus T., Moore P.L., Wibmer C.K., Tumba N.L., Werner L., Mlisana K., Sibeko S., Williamson C. (2011). The neutralization breadth of HIV-1 develops incrementally over four years and is associated with CD4+ T cell decline and high viral load during acute infection. J. Virol..

[58] Mikell I., Sather D.N., Kalams S.A., Altfeld M., Alter G., Stamatatos L. (2011). Characteristics of the earliest cross-neutralizing antibody response to HIV-1. PLoS Pathog..

[59] Moore P.L., Gray E.S., Wibmer C.K., Bhiman J.N., Nonyane M., Sheward D.J., Hermanus T., Bajimaya S., Tumba N.L., Abrahams M.R. (2012). Evolution of an HIV glycan-dependent broadly neutralizing antibody epitope through immune escape. Nat. Med..

[60] Murphy M.K., Yue L., Pan R., Boliar S., Sethi A., Tian J., Pfafferot K., Karita E., Allen S.A., Cormier E. (2013). Viral escape from neutralizing antibodies in early subtype A HIV-1 infection drives an increase in autologous neutralization breadth. PLoS Pathog..

[61] Holl T.M., Haynes B.F., Kelsoe G. (2010). Stromal cell independent B cell development *in vitro*: Generation and recovery of autoreactive clones. J. Immunol. Methods.

[62] Nojima T., Haniuda K., Moutai T., Matsudaira M., Mizokawa S., Shiratori I., Azuma T., Kitamura D. (2011). *In-vitro* derived germinal centre B cells differentially generate memory B or plasma cells *in vivo*. Nat. Commun..

[63] Liao H.X., Bonsignori M., Alam S.M., McLellan J.S., Tomaras G.D., Moody M.A., Kozink D.M., Hwang K.K., Chen X., Tsao C.Y. (2013). Vaccine induction of antibodies against a structurally heterogeneous site of immune pressure within HIV-1 envelope protein variable regions 1 and 2. Immunity.

[64] Wu X., Yang Z.Y., Li Y., Hogerkorp C.M., Schief W.R., Seaman M.S., Zhou T., Schmidt S.D., Wu L., Xu L. (2010). Rational design of envelope identifies broadly neutralizing human monoclonal antibodies to HIV-1. Science.

[65] McElrath M.J., Haynes B.F. (2010). Induction of immunity to human immunodeficiency virus type-1 by vaccination. Immunity.

[66] Burton D.R., Poignard P., Stanfield R.L., Wilson I.A. (2012). Broadly neutralizing antibodies present new prospects to counter highly antigenically diverse viruses. Science.

[67] Lingwood D., McTamney P.M., Yassine H.M., Whittle J.R., Guo X., Boyington J.C., Wei C.J., Nabel G.J. (2012). Structural and genetic basis for development of broadly neutralizing influenza antibodies. Nature.

